# Supplementary material for: The CAFA challenge reports improved protein function prediction and new functional annotations for hundreds of genes through experimental screens
Source: Genome Biol. 2019 Nov 19;20:244. doi: 10.1186/s13059-019-1835-8 (PMC6864930; doi:10.1186/s13059-019-1835-8)
Supplement: Supplementary file 1 — Additional file 1 Additional figures and tables referenced in the article. [file 13059_2019_1835_MOESM1_ESM.pdf]

Supplementary material: "The CAFA challenge reports improved protein function prediction and new functional annotations for hundreds of genes through experimental screens"

List of Figures

S1 Head-to-head comparison between top five CAFA3 versus CAFA1 methods . . . 3
S2 $F_{\max}$ curves for the top-performing methods in *partial* evaluation mode for (A) Molecular Function ontology, (B) Biological Process ontology, (C) Cellular Component ontology on *No Knowledge* benchmark. . . . 4
S3 $F_{\max}$ curves for the top-performing methods on *limited knowledge* benchmarks for (A) Molecular Function ontology, (B) Biological Process ontology, (C) Cellular Component ontology in *full* evaluation mode . . . 5
S4 Weighted precision-recall curves for the top-performing methods for (A) Molecular Function ontology, (B) Biological Process ontology, (C) Cellular Component ontology on *No Knowledge* benchmark and *full* evaluation mode . . . 6
S5 Normalized RU-MI curves for the top-performing methods for (A) Molecular Function ontology, (B) Biological Process ontology, (C) Cellular Component ontology on *No Knowledge* benchmark and *full* evaluation mode . . . 7
S6 Top 10 $F_{\max}$ in *Homo Sapiens* . . . 9
S7 Top 10 $F_{\max}$ in *Arabidopsis thaliana* . . . 10
S8 Top 10 $F_{\max}$ in *Mus musculus* . . . 11
S9 Top 10 $F_{\max}$ in *Rattus norvegicus* . . . 12
S10 Top 10 $F_{\max}$ in *Escherichia coli K12* . . . 13
S11 Top 10 $F_{\max}$ in *Drosophila melanogaster* . . . 14
S12 Top 10 $F_{\max}$ in *Dictyostelium discoideum* . . . 15
S13 Top 10 $F_{\max}$ in *Danio rerio* . . . 16
S14 Top 10 $F_{\max}$ in *Candida albicans (strain SC5314 / ATCC MYA-2876)* . . . 17
S15 Distribution of benchmark depth in leaf nodes. A leaf node is defined if any descendent nodes are not included as benchmark. . . . 21
S16 Frequency of total information content of benchmark proteins for (a) Molecular Function ontology, (b) Biological Process ontology, and (c) Cellular Component ontology. Data include all benchmark proteins and all experimentally annotated proteins at the point of benchmark collection t_1. The red point indicates the value of information content for the predicted annotation using to the Naïve model. . . . 22

List of Tables

S1 Number of experimental annotations in UniProt-GOA for biofilm formation (GO:0042710) . . 18
S2 Number of experimental annotations in UniProt-GOA for cilium or flagellum-dependent cell motility (GO:0001539) 19
S3 Number of experimental annotations in UniProt-GOA for long-term memory (GO:0007616) . 20

Contents

Mathematical definitions of protein-centric metrics **23**

List of CAFA3 Keywords **25**

Fig S1. Head-to-head comparison between top five CAFA3 versus CAFA1 methods

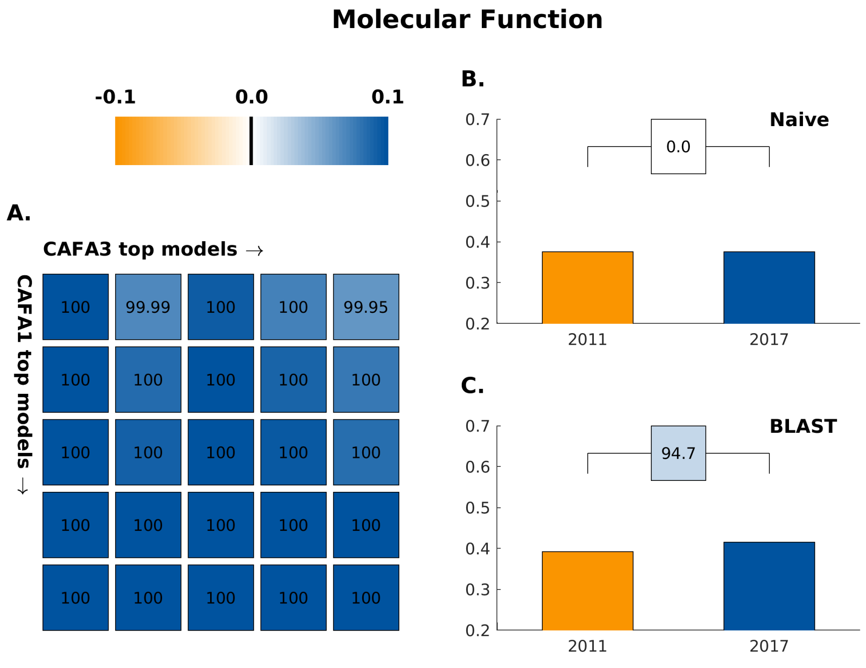

(a)

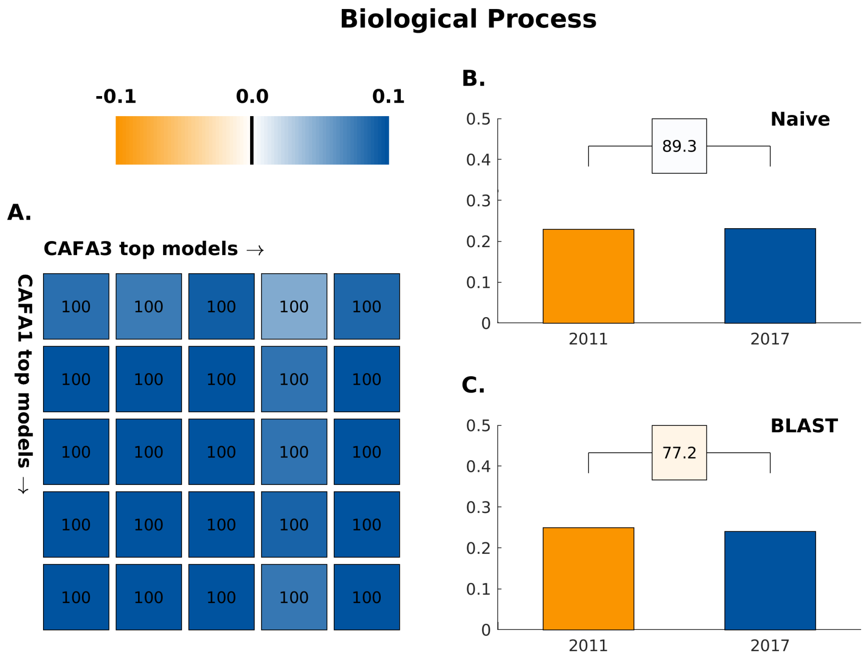

(b)

Fig S2. $F_{\max}$ curves for the top-performing methods in *partial* evaluation mode for (A) Molecular Function ontology, (B) Biological Process ontology, (C) Cellular Component ontology on *No Knowledge* benchmark.

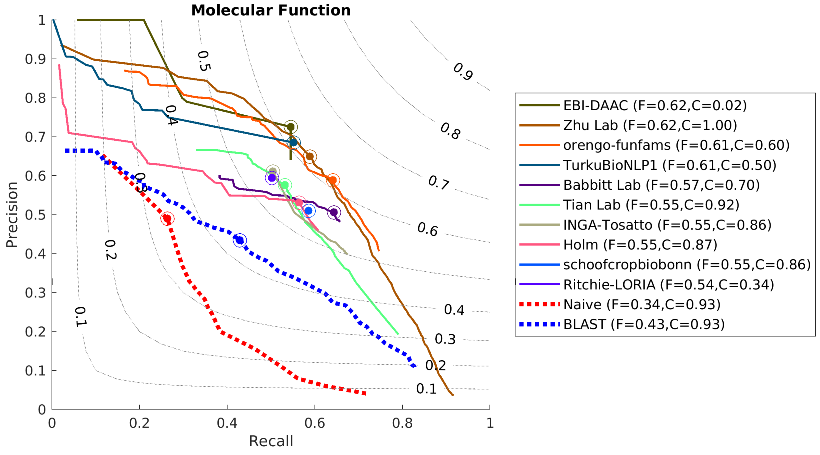

(a)

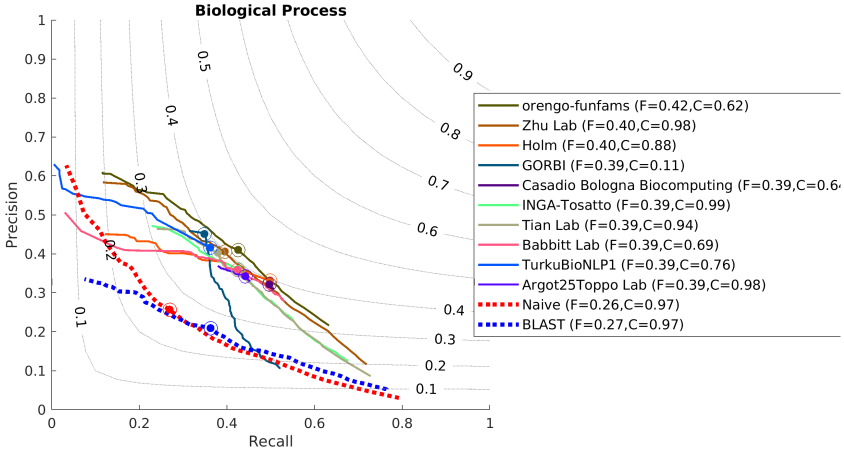

(b)

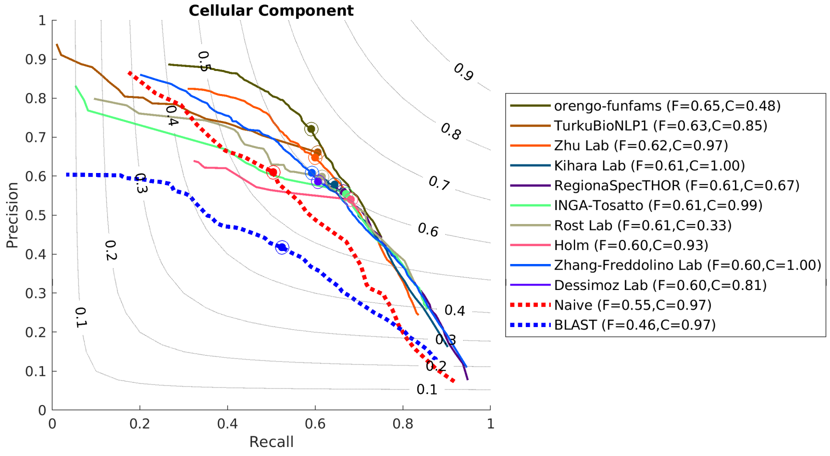

(c)

Fig S3. $F_{\max}$ curves for the top-performing methods on *limited knowledge* benchmarks for (A) Molecular Function ontology, (B) Biological Process ontology, (C) Cellular Component ontology in *full* evaluation mode

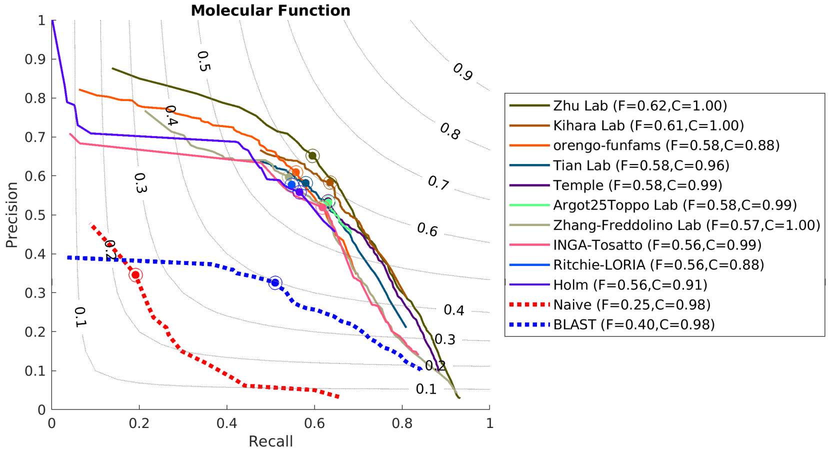

(a)

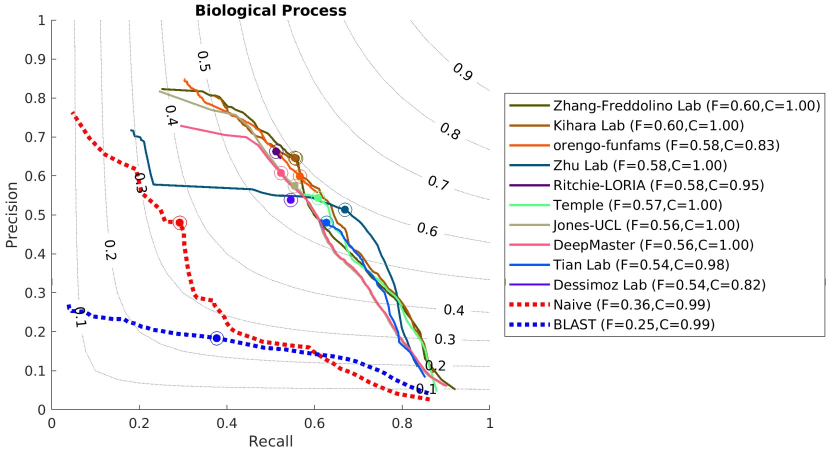

(b)

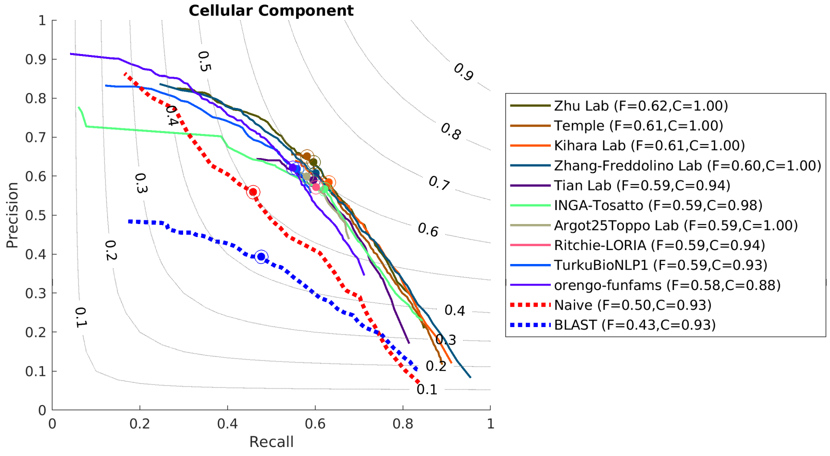

(c)

Fig S4. Weighted precision-recall curves for the top-performing methods for (A) Molecular Function ontology, (B) Biological Process ontology, (C) Cellular Component ontology on *No Knowledge* benchmark and *full* evaluation mode

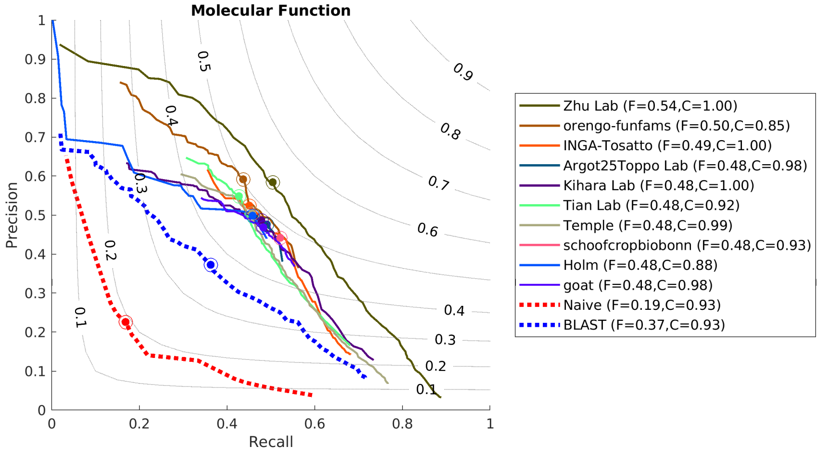

(a)

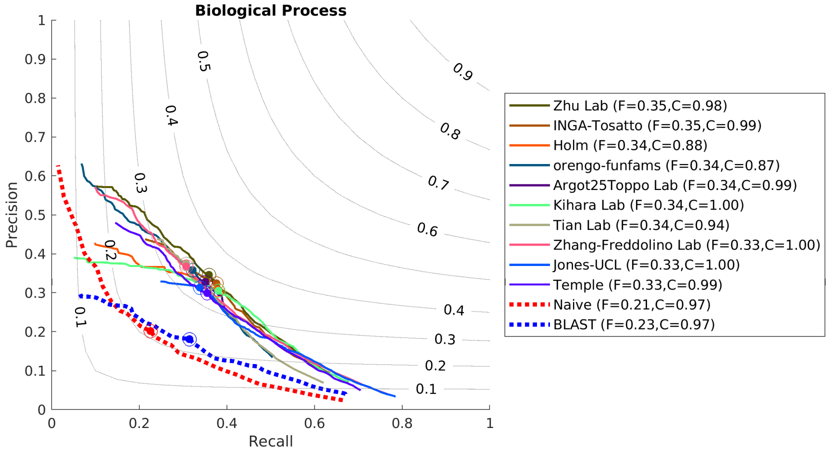

(b)

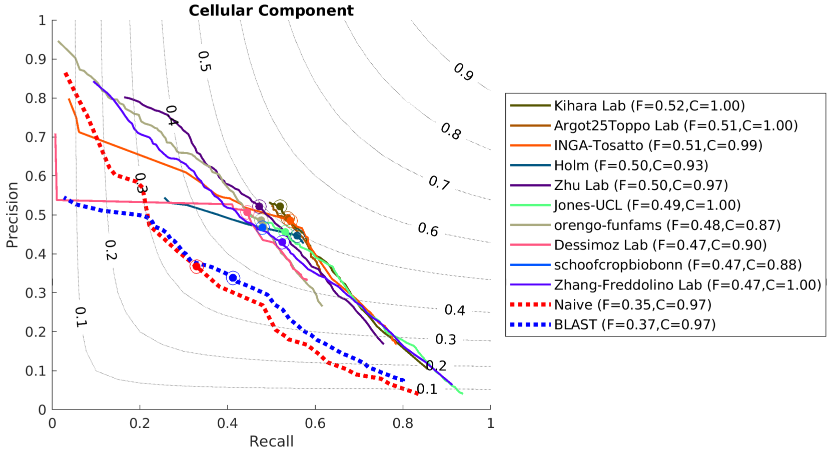

(c)

Fig S5. Normalized RU-MI curves for the top-performing methods for (A) Molecular Function ontology, (B) Biological Process ontology, (C) Cellular Component ontology on *No Knowledge* benchmark and *full* evaluation mode

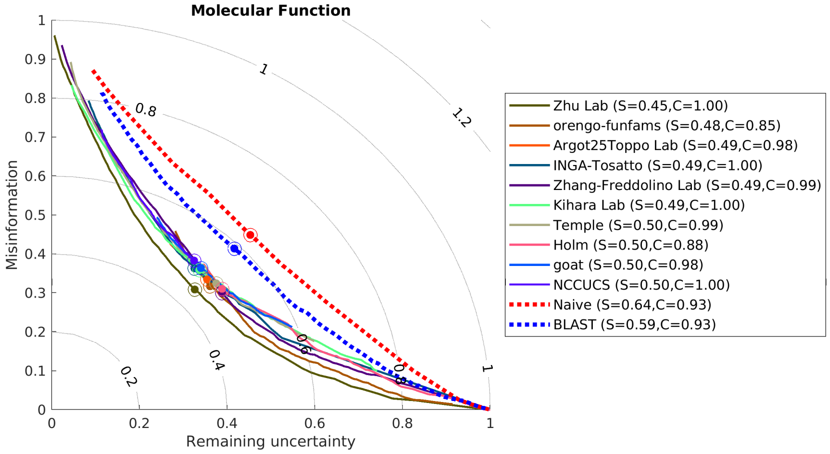

(a)

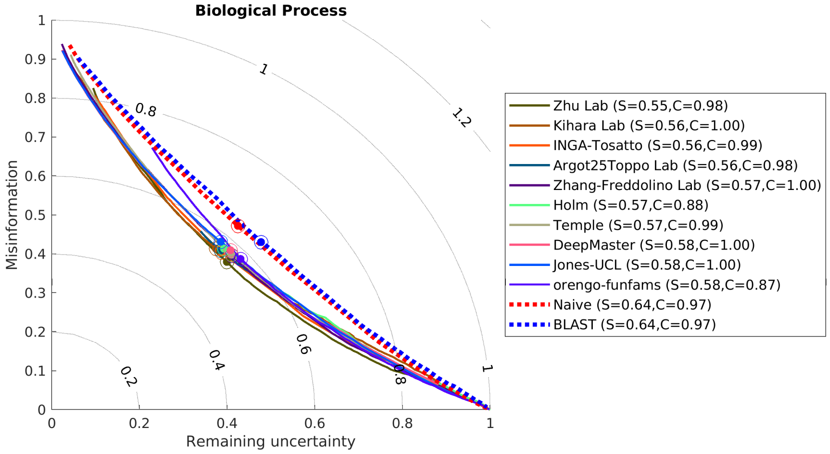

(b)

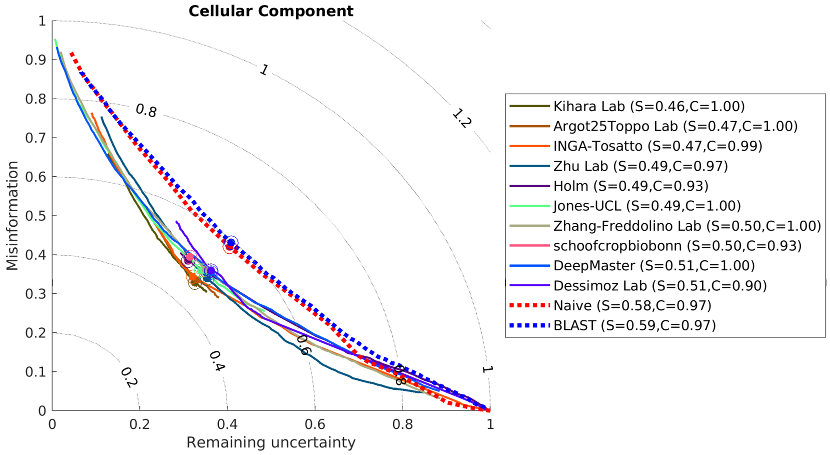

(c)

The followings figures S6, S7, S8, S9, S10, S11, S12, S13, S14 shows evaluations based on the *No Knowledge* benchmarks in the *full* evaluation mode.

Fig S6. Top 10 $F_{\max}$ in *Homo Sapiens*

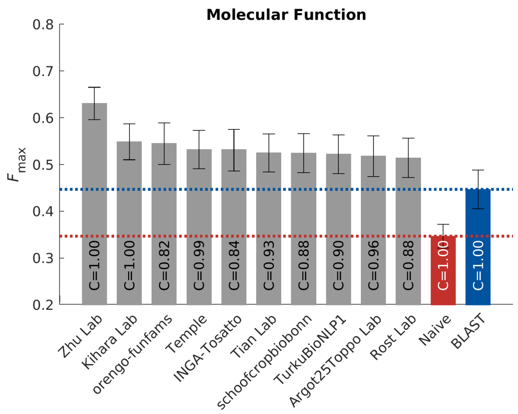

(a)

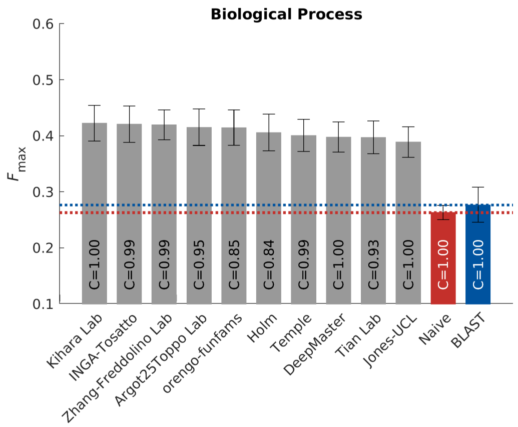

(b)

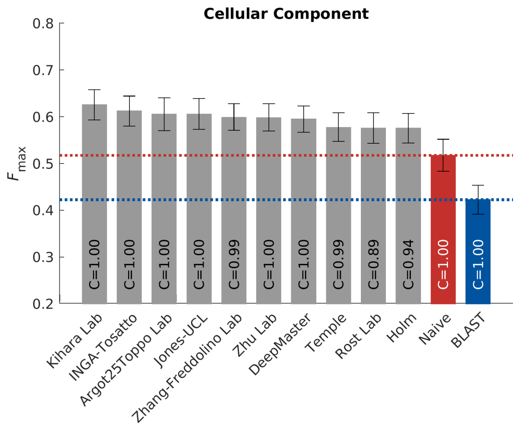

(c)

Fig S7. Top 10 $F_{\max}$ in *Arabidopsis thaliana*

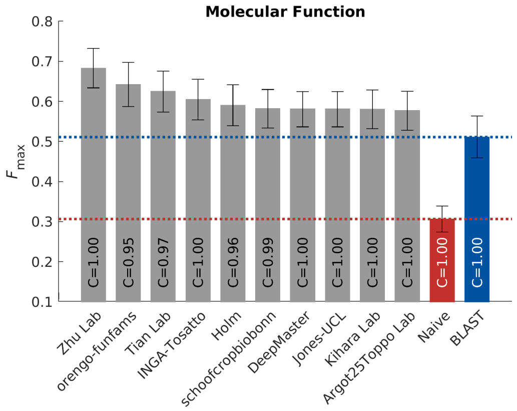

(a)

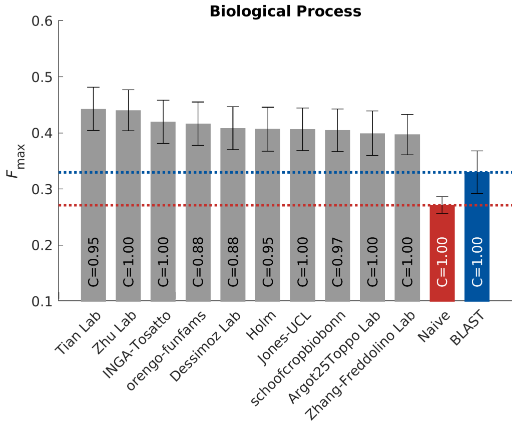

(b)

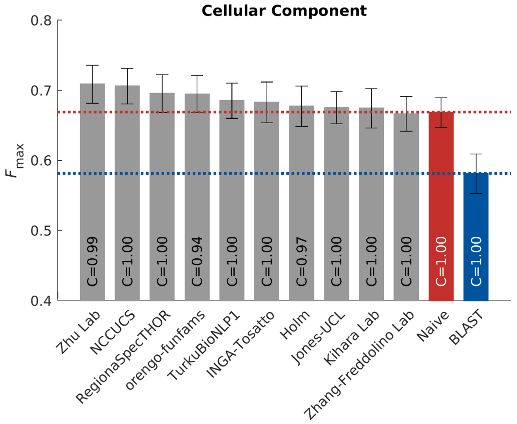

(c)

Fig S8. Top 10 F_{max} in *Mus musculus*

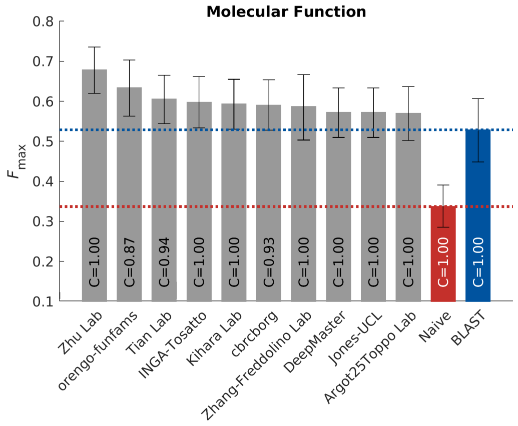

(a)

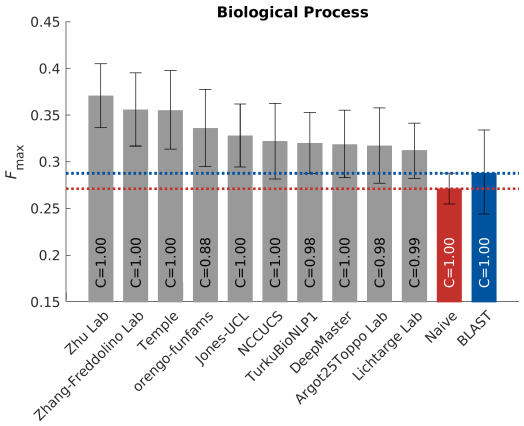

(b)

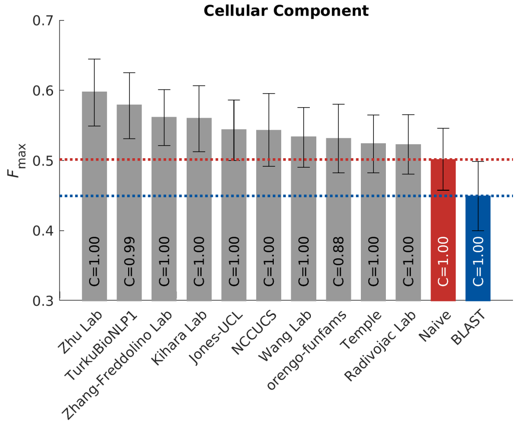

(c)

Fig S9. Top 10 $F_{\max}$ in *Rattus norvegicus*

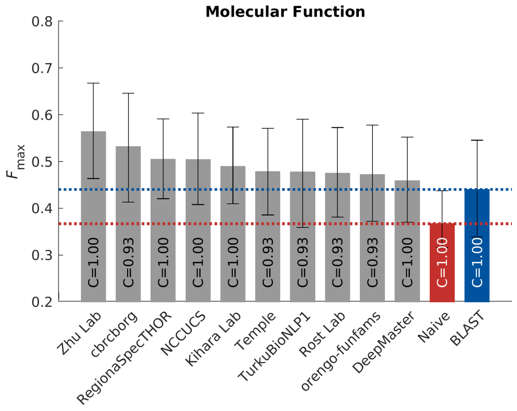

(a)

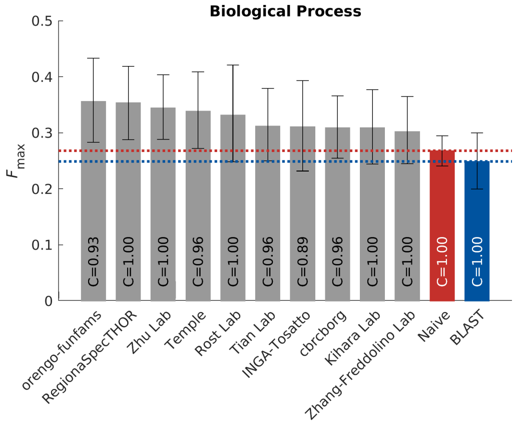

(b)

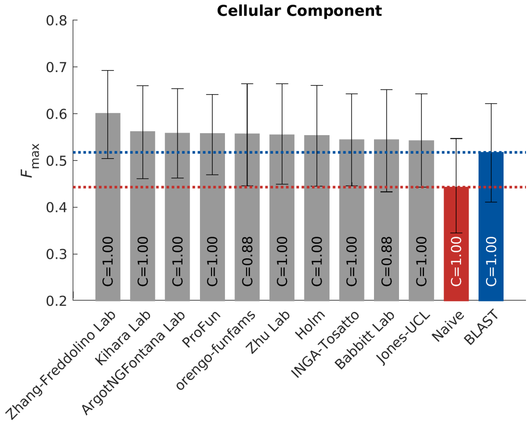

(c)

Fig S10. Top 10 $F_{\max}$ in *Escherichia coli K12*

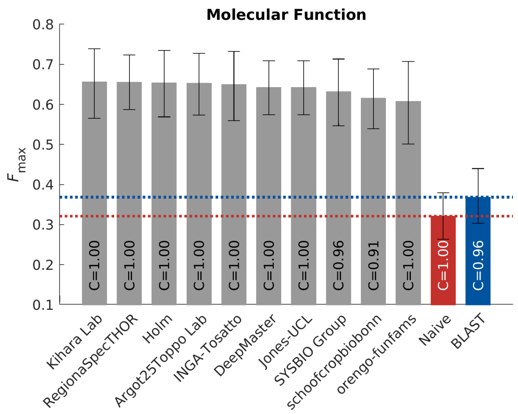

(a)

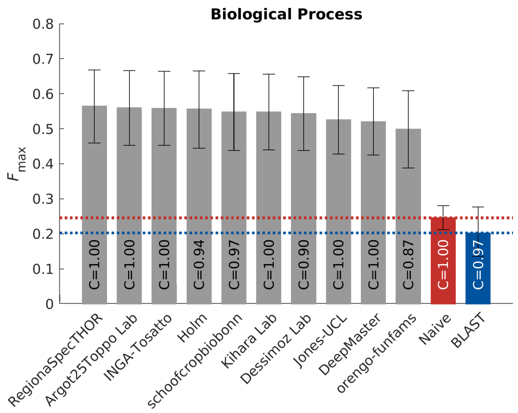

(b)

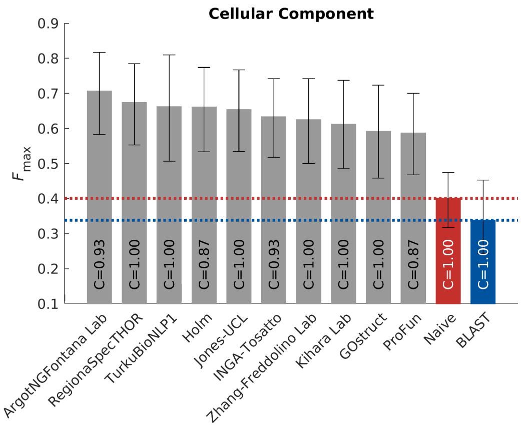

(c)

Fig S11. Top 10 $F_{\max}$ in *Drosophila melanogaster*

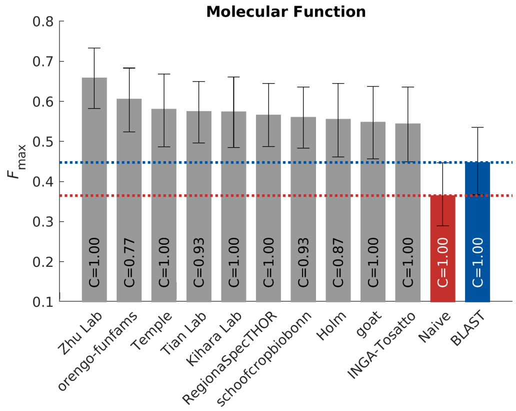

(a)

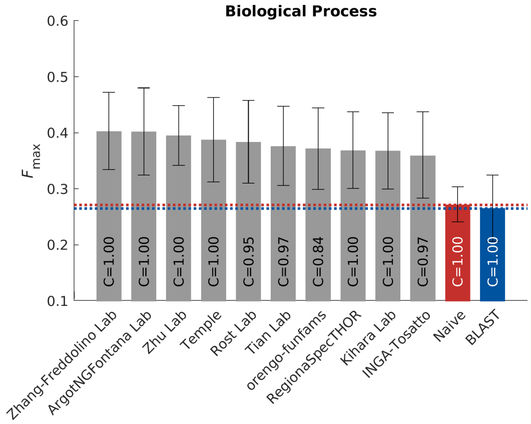

(b)

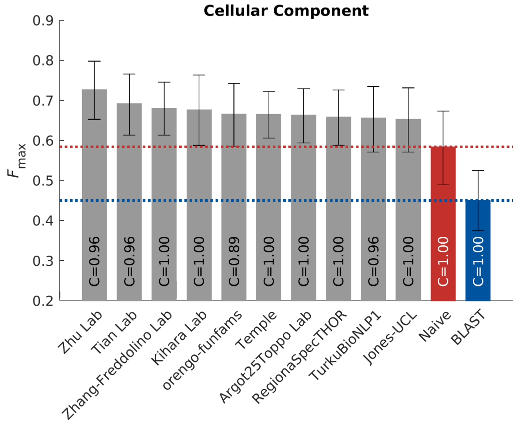

(c)

Fig S12. Top 10 $F_{\max}$ in *Dictyostelium discoideum*

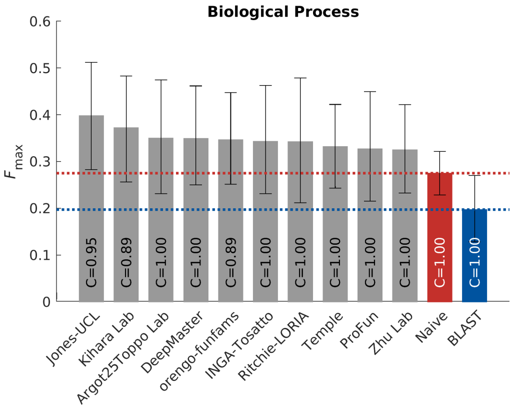

(a)

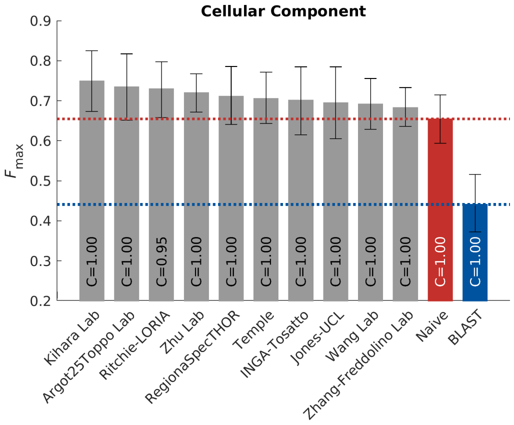

(b)

Fig S13. Top 10 $F_{\max}$ in *Danio rerio*

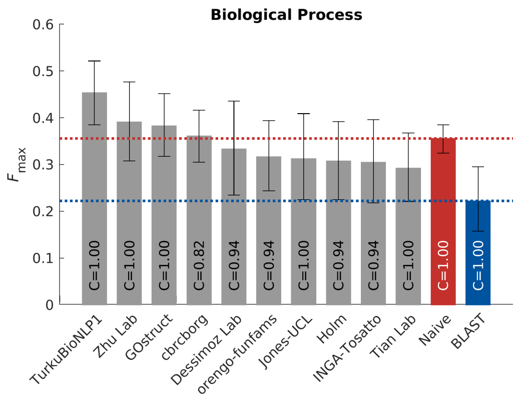

(a)

Fig S14. Top 10 $F_{\max}$ in *Candida albicans (strain SC5314 / ATCC MYA-2876)*

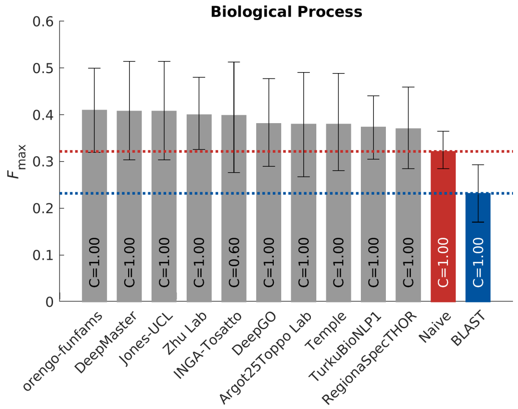

(a)

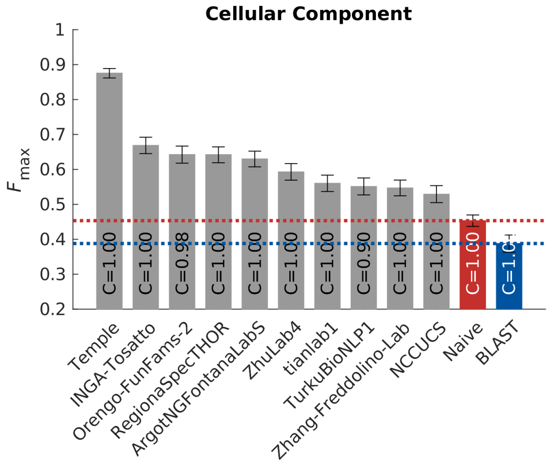

(b)

Table S1: Number of experimental annotations in UniProt-GOA for biofilm formation (GO:0042710)

Taxonomy ID	Scientific Name	Number of experimental annotations
237561	*Candida albicans* SC5314	188
208964	*Pseudomonas aeruginosa* PAO1	54
83333	*Escherichia coli* K-12	46
559292	*Saccharomyces cerevisiae* S288C	10
284593	*[Candida] glabrata* CBS 138	5
578454	*Candida parapsilosis* CDC317	4
190486	*Xanthomonas citri pv. citri* str. 306	2
290339	*Cronobacter sakazakii* ATCC BAA-894	2
216592	*Escherichia coli* 042	2
5476	*Candida albicans*	2
224308	*Bacillus subtilis subsp. subtilis* str. 168	1
1314	*Streptococcus pyogenes*	1
330879	*Aspergillus fumigatus* Af293	1
210007	*Streptococcus mutans* UA159	1
243277	*Vibrio cholerae O1 biovar El Tor* str. N16961	1
227321	*Aspergillus nidulans* FGSC A4	1
93061	S*taphylococcus aureus subsp. aureus* NCTC 8325	1
316273	*Xanthomonas campestris pv. vesicatoria* str. 85-10	1
1280	*Staphylococcus aureus*	1
100226	*Streptomyces coelicolor* A3(2)	1

Table S2: Number of experimental annotations in UniProt-GOA for cilium or flagellum-dependent cell motility (GO:0001539)

Taxonomy ID	Scientific Name	Number of experimental annotations
208964	*Pseudomonas aeruginosa* PAO1	41
3055	*Chlamydomonas reinhardtii*	40
83333	*Escherichia coli* K-12	30
185431	*Trypanosoma brucei brucei* TREU927	26
7227	*Drosophila melanogaster*	16
10090	*Mus musculus*	7
7955	*Danio rerio*	5
9606	*Homo sapiens*	4
287	*Pseudomonas aeruginosa*	4
85962	*Helicobacter pylori* 26695	3
224308	*Bacillus subtilis subsp. subtilis* str. 168	2
189518	*Leptospira interrogans serovar Lai* str. 56601	1
5664	*Leishmania major*	1
246197	*Myxococcus xanthus* DK 1622	1
9913	*Bos taurus*	1
529507	*Proteus mirabilis* HI4320	1
9615	*Canis lupus familiaris*	1
99287	*Salmonella enterica subsp. enterica serovar Typhimurium* str. LT2	1
8090	*Oryzias latipes*	1
31286	*Trypanosoma brucei rhodesiense*	1

Table S3: Number of experimental annotations in UniProt-GOA for long-term memory (GO:0007616)

Taxonomy ID	Scientific Name	Number of experimental annotations
7227	*Drosophila melanogaster*	217
10090	*Mus musculus*	23
10116	*Rattus norvegicus*	22
6239	*Caenorhabditis elegans*	13
9606	*Homo sapiens*	7
381128	*Lehmannia marginata*	1

Fig S15. Distribution of benchmark depth in leaf nodes. A leaf node is defined if any descendent nodes are not included as benchmark.

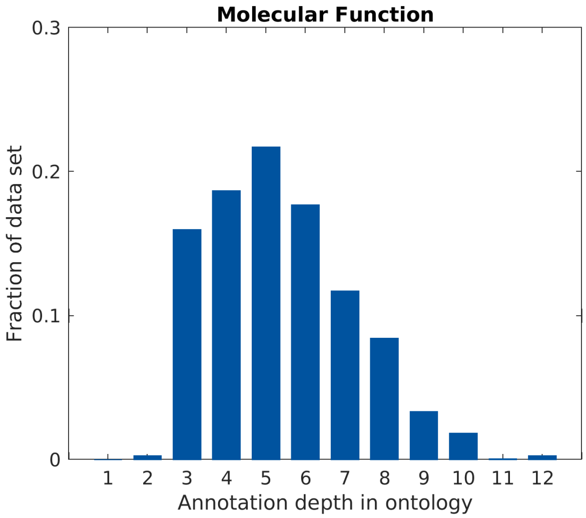

(a) After removing single protein-binding annotations

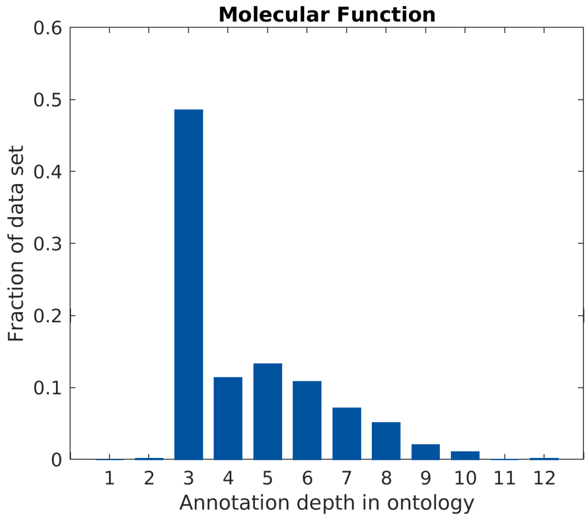

(b) Before removing single protein-binding annotations

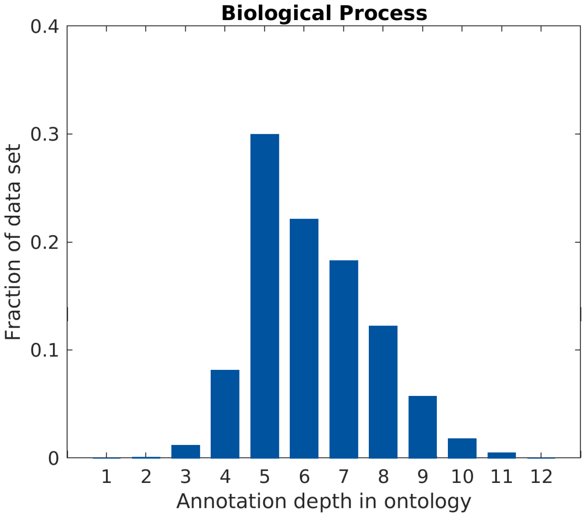

(c)

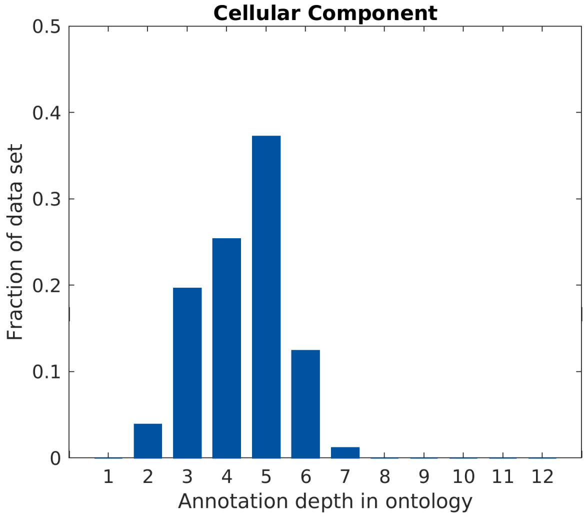

(d)

Fig S16. Frequency of total information content of benchmark proteins for (a) Molecular Function ontology, (b) Biological Process ontology, and (c) Cellular Component ontology. Data include all benchmark proteins and all experimentally annotated proteins at the point of benchmark collection t_1. The red point indicates the value of information content for the predicted annotation using to the Naïve model.

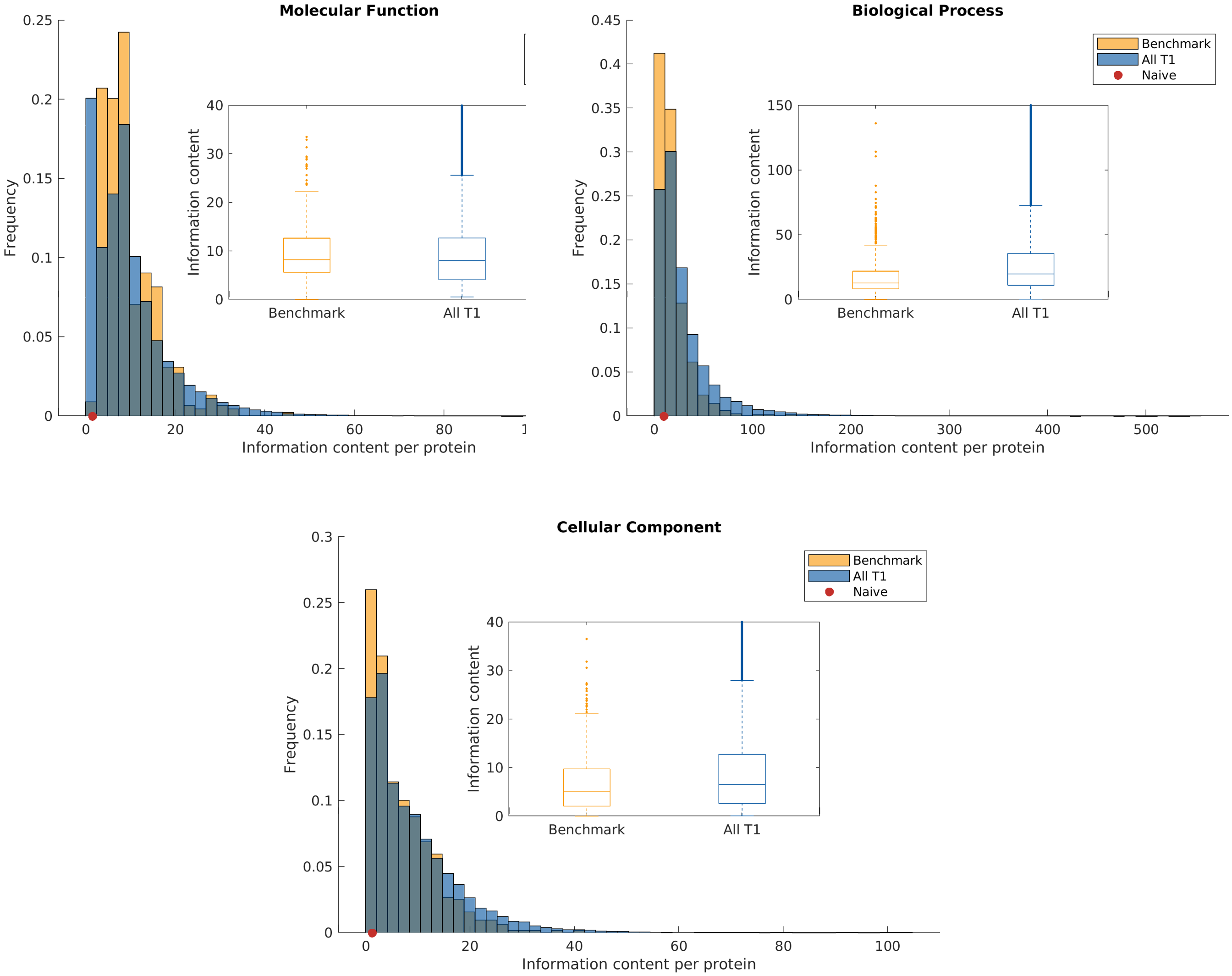

Mathematical definitions of protein-centric metrics

- Precision Recall

$$
\begin{aligned}
pr(\tau) &= \frac{1}{m(\tau)} \sum_{i=1}^{m(\tau)} \frac{\sum_f \mathbb{1}\left(f \in P_i(\tau) \wedge f \in T_i\right)}{\sum_f \mathbb{1}\left(f \in P_i(\tau)\right)}, \\
rc(\tau) &= \frac{1}{n_e} \sum_{i=1}^{n_e} \frac{\sum_f \mathbb{1}\left(f \in P_i(\tau) \wedge f \in T_i\right)}{\sum_f \mathbb{1}\left(f \in T_i\right)}, \\
F_{\max} &= \max_{\tau} \left\{ \frac{2 \cdot pr(\tau) \cdot rc(\tau)}{pr(\tau) + rc(\tau)} \right\},
\end{aligned}
$$

where $P_i(\tau)$ denotes the set of terms that have predicted scores greater than or equal to τ for a protein sequence i, T_i denotes the corresponding ground-truth set of terms for that sequence, $m(\tau)$ is the number of sequences with at least one predicted score greater than or equal to τ, $\mathbb{1}(\cdot)$ is an indicator function and n_e is the number of targets used in a particular mode of evaluation. In the full evaluation mode $n_e = n$, the number of benchmark proteins, whereas in the partial evaluation mode $n_e = m(0)$; i.e., the number of proteins which were chosen to be predicted using the particular method. For each method, we refer to $\frac{m(0)}{n}$ as the *coverage* because it provides the fraction of benchmark proteins on which the method made any predictions.

- Remaining Uncertainty and Missing Information

$$
\begin{aligned}
ru(\tau) &= \frac{1}{n_e} \sum_{i=1}^{n_e} \sum_f ic(f) \cdot \mathbb{1}\left(f \notin P_i(\tau) \wedge f \in T_i\right), \\
mi(\tau) &= \frac{1}{n_e} \sum_{i=1}^{n_e} \sum_f ic(f) \cdot \mathbb{1}\left(f \in P_i(\tau) \wedge f \notin T_i\right), \\
S_{\min} &= \min_{\tau} \left\{ \sqrt{ru(\tau)^2 + mi(\tau)^2} \right\},
\end{aligned}
$$

where $ic(f)$ is the information content of the ontology term f [**?**]. It is estimated in a maximum likelihood manner as the negative binary logarithm of the conditional probability that the term f is present in a protein's annotation given that all its parent terms are also present. Note that here, $n_e = n$ in the full evaluation mode and $n_e = m(0)$ in the partial evaluation mode applies to both ru and mi.

- Weighted Precision Recall

$$wpr(\tau) = \frac{1}{m(\tau)} \sum_{i=1}^{m(\tau)} \frac{\sum_f ic(f) \cdot \mathbb{1}\left(f \in P_i(\tau) \wedge T_i(\tau)\right)}{\sum_f ic(f) \cdot \mathbb{1}\left(f \in P_i(\tau)\right)}, \quad \text{and}$$

$$wrc(\tau) = \frac{1}{n_e} \sum_{i=1}^{n_e} \frac{\sum_f ic(f) \cdot \mathbb{1}\left(f \in P_i(\tau) \wedge T_i(\tau)\right)}{\sum_f ic(f) \cdot \mathbb{1}\left(f \in T_i(\tau)\right)},$$

where $P_i(\tau)$ is the set of predicted terms for protein i with score no less than threshold τ and T_i is the set of true terms for protein i, $m(\tau)$ is the number of sequences with at least one predicted score greater than or equal to τ, and n_e is the number of proteins used in a particular mode of evaluation. In the full evaluation mode $n_e = n$, the number of benchmark proteins, whereas in the partial evaluation mode $n_e = m(0)$.

- Normalized Remaining Uncertainty and Missing Information

$$nru(\tau) = \frac{1}{n_e} \sum_{i=1}^{n_e} \frac{\sum_f ic(f) \cdot \mathbb{1}\left(f \notin P_i(\tau) \wedge f \in T_i\right)}{\sum_f ic(f) \cdot \mathbb{1}\left(f \in P_i(\tau) \vee f \in T_i\right)}, \quad \text{and}$$

$$nmi(\tau) = \frac{1}{n_e} \sum_{i=1}^{n_e} \frac{\sum_f ic(f) \cdot \mathbb{1}\left(f \in P_i(\tau) \wedge f \notin T_i\right)}{\sum_f ic(f) \cdot \mathbb{1}\left(f \in P_i(\tau) \vee f \in T_i\right)},$$

where $P_i(\tau)$ is the set of predicted terms for protein i with score no less than threshold τ and T_i is the set of true terms for protein i, and n_e is the number of proteins used in a particular mode of evaluation. In the full evaluation mode $n_e = n$, the number of benchmark proteins, whereas in the partial evaluation mode n_e is the number of proteins that have at least one positive predicted score.

List of CAFA3 Keywords

sequence alignment, sequence-profile alignment, profile-profile alignment, phylogeny, sequence properties, physicochemical properties, predicted properties, protein interactions, gene expression, mass spectrometry, genetic interactions, protein structure, literature, genomic context, synteny, structure alignment, comparative model, predicted protein structure, de novo prediction, machine learning, genome environment, operon, ortholog, paralog, homolog, hidden Markov model, clinical data, genetic data, natural language processing, other functional information
