# Supplementary material for: The CAFA challenge reports improved protein function prediction and new functional annotations for hundreds of genes through experimental screens
Source: Genome Biol. 2019 Nov 19;20:244. doi: 10.1186/s13059-019-1835-8 (PMC6864930; doi:10.1186/s13059-019-1835-8)
Supplement: Supplementary file 2 — Additional file 2 Review History. [file 13059_2019_1835_MOESM2_ESM.docx]

Review History

**First round of review**

**Reviewer 1**

**Are you able to assess all statistics in the manuscript, including the appropriateness of statistical tests used?**

Yes, and I have assessed the statistics in my report.

**Comments to author:**

This manuscript presents in detail the results of the CAFA3 challenge of functional annotation, as well as CAFAπ, which followed with new process-centric experimental data. The results are interesting and relevant to the broader community. Even though progress has not been large from CAFA2 to CAFA3, there is one clear winner, a new method, and the study provides additional insight into which methods work and on future directions of research.

The statistical analyses follow those of CAFA2, and are appropriate.

While the results are very interesting, the text and figures are difficult to follow, maybe because of "writing by committee", and maybe because of rushing to reach the special issue deadline. In addition to the presentation which should be improved, I have a few suggestions to improve the manuscript.

Detailed major comments:

lines 63-64: "BLAST-based annotation transfer, tells a contrasting tale between ontologies" Not really; change is slight in all ontologies. It reaches "significance" for MFO, but maybe because of dataset size? In any case, significance without effect size should not be highlighted.

The authors conclude from different results on the three ontologies of the GO that "the ontologies are in different annotation states and should not be treated as a whole". Wouldn't similar conclusions be reached if subsets of these ontologies were considered (e.g. binding vs. catalytic activity etc)? How do we know what is the natural level to treat functional annotations?

p. 9, among the explanation for the low improvement from CAFA2 to CAFA3, a possibility is a plateau in the power of the approaches used so far; this possibility is supported by the results of expression data, which indicate sources of information which have been poorly tapped so far. There might also be totally new approaches which would lead to large improvements, but have not yet been explored.

Given the ubiquity of use of Blast first hit for annotation, I think that the message that it is less good than "naive" (i.e. random) annotation for 2 of 3 GO ontologies should be emphasized.

Most of the paper emphasizes the ranking by Fmax, while differences between methods are less clear using Smin. It could be argued that the latter is more relevant. Could the authors please justify their choice to emphasize Fmax?

Comparison of eukaryotes and prokaryotes:
- Why use this terminology, rather than eukaryotes and eubacteria (and eventually archeae, but do you have any in the dataset?)?
- This should not be presented in terms of comparing "species", as it is comparing two very large and heterogeneous groups of species.

Section "Long-term memory in D. melanogaster" is too short, and should be expanded to be useful.

Why propose to include a "baseline expression-based method" in future CAFA evaluations, rather than including it in this report?

Please provide the reference everytime you meantion a method, especially the new top method of the Zhu lab. For the Zhu method, please present and discuss it at least briefly in the Results and Discussion.

Many terms and concepts are not defined, or not clearly, and many figures are lacking clear legends:
- line 288: "the Smin is based the RU-MI curve", a definition of RU-MI should be given at this place.
- Smin should be briefly defined at first use in the Results.
- Fig 1, what is the number in a box between the two years in B and C?
- Fig 4, the point on the lines is not defined as far as I can find.
- Fig 5, please remind what is Smin, noting the lower is better (unlike Fmax which is in all the previous figures). Please give some indication on the interpretation of the y-axis scale.
- AUROC is never defined (I know it's area under ROC, but it's not a very common use in biology and should be specified)

All barplots should start at 0, or alternatively another visualisation should be preferred. In the many figure where Fmax is the y-axis and coverage is presented as a number within the bar, a 2D plot with coverage in x and Fmax in y could be used.

Fig 6 is difficult to read. I suggest a scatter plot with eukaryotic scores on one axis and prokaryotic (or bacterial) scores on the other.

Fig 7 is almost impossible to read. I suggest heat maps of Euclidian scores between methods.
Also, why a cut-off of 0.07?

I found Fig 8 very interesting. I suggest ranking keywords by weighted frequency though.
It would also be very interesting to show which keywords differ the most in weighted frequency between the 3 ontologies.

Fig 9, why not include a horizontal dotted line for the baseline results? There could be one line for the worst and one for the best baseline.
The legend should specify what the color code (dark blue, light blue) means.

What is the justification for the rule that "Each Principle Investigator is allowed to head multiple teams, but each member can only belong to one team"? (especially the last part)

Minor comments:

line 76 "the annotation databases remain sparsely populated" should be "the annotation databases remain too sparsely populated".
line 155, it is not (all) "UniProt" but "Swiss-Prot" which is curated.
Fig 1 would be clearer with method names specified.
legend of Fig 2, what does this mean? "CAFA1 methods were ranked but not displayed"

**Reviewer 2**

**Are you able to assess all statistics in the manuscript, including the appropriateness of statistical tests used?**

Yes, and I have assessed the statistics in my report.

**Comments to author:**

CAFA is an open challenge for predicting protein annotation and function. The current manuscript is the third iteration of CAFA, and this version is not only incorporates computational predictions, but follow up experiments in C. albicans, P. aureginosa and D. melanogaster.  The goal of the CAFA challenge and the CAFA community is to close the gap between data being generated and translating these data into biological knowledge, through functional annotation. The paper offers a short summary of CAFA1 and 2 and nicely motivates CAFA3. The team also provides an evaluation of how predictions have progressed over time, with some interesting observations, such as the baseline model not improving over time, that sequence similarity methods don't really benefit from a larger training set, the variation that is seen in performance on different GO classes, and the apparent plateauing of performance of the top methods in CAFA3. An ensemble method is the top performing method, with machine learning and sequencing based method being the most popular methods. 

A nice addition to CAFA3 is the experimental data generated and CAFA-pi.  The main conclusion from these experiments, in addition to generating new functionally and experimentally supported annotations, is that sequence only methods are not sufficient. It is hard to compare the Drosophila results with only 29 target genes tested to the genome-wide screens in microbes. 

Overall, the CAFA team has done a nice job at providing a rich and useful benchmarked analysis of functional annotation prediction. The methods and scoring approaches are well established and the results are robust. The data should be shared with the Bioinformatics community and it shows that over the 3 iterations of CAFA the manuscript writing process is mature.  There are no concerns from this reviewer and I support publication as is. 

From my reading of the results from CAFA1-3, it appears there are a group of participants that apply the same or slightly modified methods. Given the lack of major improvement from 2 to 3, the authors might consider ways to stimulate method innovation in CAFA4 if that is planned.  The same methods in CAFA3 could be automatically applied in CAFA4 with the goal to stimulate novelty in method development if the authors believe there is still room for improvement.

**Reviewer 3**

**Are you able to assess all statistics in the manuscript, including the appropriateness of statistical tests used?**

No, I do not feel adequately qualified to assess the statistics.

**Comments to author:**

The manuscript reports the CAFA community to improve protein functional annotation, computational function prediction, which results in new functional annotations for more than 1000 genes. Overall, these are interesting work which provides a useful resource for  the  scientific community in the field. Most of the claims of the research are well supported by the experimental evidence. One of the most important parts of this study is the whole genome mutants screen, the methods used in the screening process is well described and repeatable by the groups of scientists independently .  
To perform the functional verification, the authors employed mutant libraries from  a few species, and evaluated the phenotype. The design of the experiment and results seems pretty solid, however, my concern is that those verified true/false annotation terms should be clarified within the context of the tested species and experimental conditions (temperature, medium, etc.). In table 1 as instance, "of the 2063 proteins that we did not find to be associated with biofilm formation, 29 were annotated to the term in the GOA database". Those 29 genes were not related to biofilm formation in Candida albicans, could be different in other species, in other conditions? As it is common that homologous genes can function distinctly in different species. Please modify the interpretation accordingly to clarify this point.

Line 186:"245 were required for the formation of wrinkled colony biofilm formation", is another example of my next concern. My understanding is the phenotypes observed in those tests  are broad, like biofilm formation here,  I believe part of the annotated genes may affect the fitness of the species and decrease the ability to form biofilms, rather than correlating to biofilm formation directly. A precise rephrasing may be necessary to improve the intended point being made.

**Authors Response**

**Point-by-point responses to the reviewers’ comments:**

Comment

*Response*

Reviewer #1: This manuscript presents in detail the results of the CAFA3 challenge of functional annotation, as well as CAFA-pi, which followed with new process-centric experimental data. The results are interesting and relevant to the broader community. Even though progress has not been large from CAFA2 to CAFA3, there is one clear winner, a new method, and the study provides additional insight into which methods work and on future directions of research. 

The statistical analyses follow those of CAFA2, and are appropriate. 

While the results are very interesting, the text and figures are difficult to follow, maybe because of "writing by committee", and maybe because of rushing to reach the special issue deadline. In addition to the presentation which should be improved, I have a few suggestions to improve the manuscript. 

Detailed major comments: 

Q: lines 63-64: "BLAST-based annotation transfer, tells a contrasting tale between ontologies" Not really; change is slight in all ontologies. It reaches "significance" for MFO, but maybe because of dataset size? In any case, significance without effect size should not be highlighted. 

*A: We agree with the reviewer that the conclusion we provide does not stem from this finding. We have therefore deleted this sentence.* 


Q: The authors conclude from different results on the three ontologies of the GO that "the ontologies are in different annotation states and should not be treated as a whole". Wouldn't similar conclusions be reached if subsets of these ontologies were considered (e.g. binding vs. catalytic activity etc)? How do we know what is the natural level to treat functional annotations? 

*A: Figures S15 and S16 show that different ontologies have different distributions of depth and information content. We added a comment in the captions to clarify that.* 

Q: p. 9, among the explanation for the low improvement from CAFA2 to CAFA3, a possibility is a plateau in the power of the approaches used so far; this possibility is supported by the results of expression data, which indicate sources of information which have been poorly tapped so far. There might also be totally new approaches which would lead to large improvements, but have not yet been explored. 

*A: That is indeed a highly plausible hypothesis, which we hope to test in future CAFA challenges. As suggested, we have added text that highlights the need for exploring the use of non-sequence data, such as expression data: lines 468-478 “Interestingly, the top performing CAFA3 method, which consistently outperformed methods from all past CAFAs in the major categories (GOLabeler), which uses five component classifiers, trained from different features, including GO term frequency, sequence alignment, amino acid trigram, domains and motifs, and biophysical properties. It best performs in the Molecular Function Ontology, where apparently sequence features perform best.Another method which did not compete in CAFA3 yet seems to perform well under CAFA parameters is NetGO, which utilizes information from STRING, a network association database in addition to sequence information. Taken together with the strong predictive performance of mRNA co-expression data) leads us to hypothesize that including more varied sources of data can lead to additional large improvements in protein function prediction. We are looking forward to testing this hypothesis in future CAFA challenges.”* 


Q: Given the ubiquity of use of Blast first hit for annotation, I think that the message that it is less good than "naive" (i.e. random) annotation for 2 of 3 GO ontologies should be emphasized. 

*A: We would like to clarify that Naive-based annotation is not random annotation, but rather annotation based on the prior frequency of terms in the corpus: annotation is assigned with a confidence that is based on that frequency. So, for example, if 30% of the terms in the corpus are “protein binding”, and 10% are annotated “Protein kinase activity” all proteins in the benchmark will be annotated by Naive with the term “protein binding” and a confidence value of 0.3, and the term “Protein kinase activity” with a confidence of 0.1. When Naive is perrfoming better than BLAST, this typically indicates a strong bias in the benchmark data, and / or a problem with the ontology construction (as is in CCO).* 

Q: Most of the paper emphasizes the ranking by Fmax, while differences between methods are less clear using Smin. It could be argued that the latter is more relevant. Could the authors please justify their choice to emphasize Fmax? 

*A: Briefly, Fmax is more interpretable. However, the use of two metrics provides a better picture of the performance of the models, that cannot be assessed by a single metric. We have added the following clarification: “It should be noted that CAFA uses both Fmax and Smin. Fmax's strength lies in its interpretability, as it is simply the maximum F1 given for each model. At the same time, precision/recall based assessment does not capture the differences in information content between different GO terms. For that reason, we also use the Smin score which incorporates information content, but is somewhat less interpretable than Fmax. Additionally, since information content of a given GO term (-log(Pi)) is derived from its frequency in the corpus (Pi), it is somewhat malleable depending on the corpus from which it is derived. We therefore use both measures for scoring, to achieve a more comprehensive picture of the models' performance.” (lines 479-487).* 

Q: Comparison of eukaryotes and prokaryotes: 
- Why use this terminology, rather than eukaryotes and eubacteria (and eventually archeae, but do you have any in the dataset?)? 
- This should not be presented in terms of comparing "species", as it is comparing two very large and heterogeneous groups of species. 
 *A: We have now corrected to “eukarya” and “bacteria”, and removed the “species” reference.* 

Q: Section "Long-term memory in D. melanogaster" is too short, and should be expanded to be useful. 

*A: We have added the following text to clarify (P. 15 lines 294-300). 
“We performed RNAi experiments in Drosophila melanogaster to assess whether 29 target genes were associated with long-term memory (GO:0007616). Briefly, flies were exposed to wasps, which triggers a behavior that causes females to lay fewer eggs. The acute response is measured until 24h post-exposure, and the long-term response is measured at 24h to 48h post-exposure. RNAi was used to interfere with the expression of the 29 target genes in the mushroom body, a region of the fly brain associated with memory. Using this assay, we identified 3 genes involved in perception of wasp exposure, and 12 genes involved in long-term memory. For details on the target selection and fly assay, see \cite{kacsoh2019new}”.* 


Q: Why propose to include a "baseline expression-based method" in future CAFA evaluations, rather than including it in this report? 

*A: The reason is that we do not have RNA-Seq or other RNA expression level data for this challenge.* 

Q: Please provide the reference every time you mention a method, especially the new top method of the Zhu lab. For the Zhu method, please present and discuss it at least briefly in the Results and Discussion. 

*A: We leave this decision to the editor. We believe that one citation per method is enough, as is common in most scientific writing. As we do not want to “play favorites”, we leave it to the reader to further read on any of the methods cited in this work.* 


Q: Many terms and concepts are not defined, or not clearly, and many figures are lacking clear legends: 
- line 288: "the Smin is based the RU-MI curve", a definition of RU-MI should be given at this place. 

*A: We have added the following sentences to explain the Smin metric: ‘“The Fmax based on the precision-recall curve (Figure 3), while the Smin is based the RU-MI curve (Figure 4), where S stands for semantic distance. The shortest semantic distance across all thresholds is used as the Smin metric. The RU-MI curve takes into account the information content of each GO term in addition to counting the number of true positives, false positives, etc. See pages 22 and 23 of Supplemental Materials for their mathematical definitions.”* 

Q: - Smin should be briefly defined at first use in the Results. 

*A: A brief explanation of the idea behind Smin can be found in the Results section at line 138 through line 141, as well as the caption of Figure 4.* 

Q: - Fig 1, what is the number in a box between the two years in B and C? 

*A: The number indicates the percentage of wins in 10,000 bootstrapped iterations comparing Fmax performance between the baseline method of the two years. We have edited the figure caption to include this information.* 

Q: - Fig 4, the point on the lines is not defined as far as I can find. 
 *A: The point is the specific Precision-Recall (RU-MI) value pair where maximum (minimum) F score (Semantic distance) is achieved. We have edited the figure caption to include this information.* 

Q: - Fig 5, please remind what is Smin, noting the lower is better (unlike Fmax which is in all the previous figures). Please give some indication on the interpretation of the y-axis scale. 
 *A: We have edited the figure caption to include a brief description of the RU-MI curve and the Smin metric, emphasizing* *that “The perfect prediction should have Smin= 0, at the bottom left corner of the plot.”* 

Q: - AUROC is never defined (I know it's area under ROC, but it's not a very common use in biology and should be specified) 
 *A: The following description of the ROC curve and the Area Under Curve metric has been added to line 235 through line 239. “We used Receiver Operating Characteristic (ROC) curves to measure the prediction accuracy. Area under ROC curves (AUROC) was used to compare the performance. AUROC is a common accuracy measure for classification problems where it evaluates how good a model is at distinguishing between the positive and negative classes.”* 


Q: All barplots should start at 0, or alternatively another visualisation should be preferred. In the many figure where Fmax is the y-axis and coverage is presented as a number within the bar, a 2D plot with coverage in x and Fmax in y could be used. 
 *A: We have edited the figures so that most of the barplots start at 0. In one case (Fig 4) we left the y-axis as is. The comparison between methods in the same ontology the bar plot is more important than between ontologies. Starting all at the same level would hamper visualization.* 

Q: Fig 6 is difficult to read. I suggest a scatter plot with eukaryotic scores on one axis and prokaryotic (or bacterial) scores on the other. 
 *A: Now Figure 5. We cannot, unfortunately, perform this suggestion as different teams provided the results for bacteria and eukaryotes, so they are not comparable on 2 axes.* 

Q: Fig 7 is almost impossible to read. I suggest heat maps of Euclidian scores between methods. 
Also, why a cut-off of 0.07? 
 *A: We have replaced the network visualization to heatmaps of Euclidian distances. (Now Figure 6). Thank you for this suggestion, the visualization has improved considerably.* 

Q: I found Fig 8 very interesting. I suggest ranking keywords by weighted frequency though. 
It would also be very interesting to show which keywords differ the most in weighted frequency between the 3 ontologies. 
 *A: We have updated this figure to ranking by weighted frequency, and added another panel to show the five keywords that differ the most in each ontology.* 

Q: Fig 9, why not include a horizontal dotted line for the baseline results? There could be one line for the worst and one for the best baseline. 
The legend should specify what the color code (dark blue, light blue) means. 
 *A: We have added a horizontal dotted line for the best-performing baseline result. We have edited the figure caption to explain the color code.* 

Q: What is the justification for the rule that "Each Principle Investigator is allowed to head multiple teams, but each member can only belong to one team"? (especially the last part) 
 *A: The rationale is to avoid abuse of the competition aspect of CAFA, and to avoid overwhelming the assessors with several “teams”, all from the same lab with the same people, using slight variations of the methods in an attempt to secure a good score simply by trial-and error, and flooding the system.* 

Minor comments: 

Q: line 76 "the annotation databases remain sparsely populated" should be "the annotation databases remain too sparsely populated". 
 *A: Fixed.* 

Q: line 155, it is not (all) "UniProt" but "Swiss-Prot" which is curated. 
 *A: Fixed.* 

Q: Fig 1 would be clearer with method names specified. 
 *A: We have tried that, but it created needless clutter. The main focus here is to show rankings over the three challenges, and not the individual methods.* 

Q: legend of Fig 2, what does this mean? "CAFA1 methods were ranked but not displayed" 
 *A: We have now fixed the test to read: “CAFA1 methods were ranked but since none made to the top 12 methods of all three CAFA challenges, they were not displayed.”* 


Reviewer #2: CAFA is an open challenge for predicting protein annotation and function. The current manuscript is the third iteration of CAFA, and this version is not only incorporates computational predictions, but follow up experiments in C. albicans, P. aureginosa and D. melanogaster. The goal of the CAFA challenge and the CAFA community is to close the gap between data being generated and translating these data into biological knowledge, through functional annotation. The paper offers a short summary of CAFA1 and 2 and nicely motivates CAFA3. The team also provides an evaluation of how predictions have progressed over time, with some interesting observations, such as the baseline model not improving over time, that sequence similarity methods don't really benefit from a larger training set, the variation that is seen in performance on different GO classes, and the apparent plateauing of performance of the top methods in CAFA3. An ensemble method is the top performing 
method, with machine learning and sequencing based method being the most popular methods. 

A nice addition to CAFA3 is the experimental data generated and CAFA-pi. The main conclusion from these experiments, in addition to generating new functionally and experimentally supported annotations, is that sequence only methods are not sufficient. It is hard to compare the Drosophila results with only 29 target genes tested to the genome-wide screens in microbes. 

Overall, the CAFA team has done a nice job at providing a rich and useful benchmarked analysis of functional annotation prediction. The methods and scoring approaches are well established and the results are robust. The data should be shared with the Bioinformatics community and it shows that over the 3 iterations of CAFA the manuscript writing process is mature. There are no concerns from this reviewer and I support publication as is. 

From my reading of the results from CAFA1-3, it appears there are a group of participants that apply the same or slightly modified methods. Given the lack of major improvement from 2 to 3, the authors might consider ways to stimulate method innovation in CAFA4 if that is planned. The same methods in CAFA3 could be automatically applied in CAFA4 with the goal to stimulate novelty in method development if the authors believe there is still room for improvement. 
 *A: We thank the reviewer for these comments. We do intend to provide additional non-sequence data for the CAFA4 challenge, which will hopefully stimulate the development and use of methods that do not use sequence data only.* 


Reviewer #3: The manuscript reports the CAFA community to improve protein functional annotation, computational function prediction, which results in new functional annotations for more than 1000 genes. Overall, these are interesting work which provides a useful resource for the scientific community in the field. Most of the claims of the research are well supported by the experimental evidence. One of the most important parts of this study is the whole genome mutants screen, the methods used in the screening process is well described and repeatable by the groups of scientists independently . 

Q: To perform the functional verification, the authors employed mutant libraries from a few species, and evaluated the phenotype. The design of the experiment and results seems pretty solid, however, my concern is that those verified true/false annotation terms should be clarified within the context of the tested species and experimental conditions (temperature, medium, etc.). In table 1 as instance, "of the 2063 proteins that we did not find to be associated with biofilm formation, 29 were annotated to the term in the GOA database". Those 29 genes were not related to biofilm formation in Candida albicans, could be different in other species, in other conditions? As it is common that homologous genes can function distinctly in different species. Please modify the interpretation accordingly to clarify this point. 
 *A: Indeed, orthologs in other species may have different functions. We have now clarified that the findings apply only to C. albicans. Page 12, lines 233-235: “Of the 2063 proteins that we did not find to be associated with biofilm formation, 29 were annotated with the term in the GOA database in C. albicans.''* 

Q: Line 186:"245 were required for the formation of wrinkled colony biofilm formation", is another example of my next concern. My understanding is the phenotypes observed in those tests are broad, like biofilm formation here, I believe part of the annotated genes may affect the fitness of the species and decrease the ability to form biofilms, rather than correlating to biofilm formation directly. A precise rephrasing may be necessary to improve the intended point being made. 
 *A: Wrinkled colony formation serves as an accepted indicator of biofilm formation in a variety of microbes. The annotated genes may, indeed, be less, or more “upstream” to the biofilm formation phenotype, yet at the end they do affect biofilm formation. As the reviewer states, these deletion mutants do decrease the ability to form biofilms. Since the only concern we have here is with the biofilm phenotype, then this is the way we left it. Those genes may and many probably do have other functions, but for the purposes of this study we are only concerned with the wrinkled colony phenotype as an indicator of biofilm formation.*
